# Comparative Cytocompatibility and Morphological Assessment of Silver Nanoparticle Formulations in Human Dental Pulp Stem Cells

**DOI:** 10.3390/dj14100682

**Published:** 2026-10-19

**Authors:** Ángeles Manzanera-Mondéjar, Julia Guerrero-Gironés, Berta Rivas-Mundiña, David García-Bernal, Sergio López-García

**Affiliations:** 1 Department of Dermatology, Stomatology, Radiology and Physical Medicine, Faculty of Medicine, Morales Meseguer Hospital, University of Murcia, 30008 Murcia, Spain; angeles.manzanera@um.es (Á.M.-M.); slg5@um.es (S.L.-G.); 2 Medical-Surgical Dentistry Research Group (OMEQUI), Health Research Institute of Santiago de Compostela (IDIS), University of Santiago de Compostela (USC), 15782 Santiago de Compostela, Spain; berta.rivas@usc.es; 3 Biomedical Research Institute of Murcia (IMIB), Department of Biochemistry, Molecular Biology B and Immunology, Faculty of Medicine, University of Murcia, 30120 Murcia, Spain; david.garcia23@um.es

**Keywords:** silver nanoparticles, human dental pulp stem cells, cytocompatibility, fluorescence microscopy, cytoskeleton

## Abstract

**Objectives:** This in vitro study evaluated the cytocompatibility and cellular response of human dental pulp stem cells (hDPSCs) exposed to two experimental polyethyleneimine/polyvinylpyrrolidone-stabilized silver nanoparticle formulations, MG-85 and MG-89. **Methods**: hDPSCs isolated from human third molars were treated with serial concentrations of both formulations. Cell metabolic activity and half-maximal inhibitory concentration (IC_50_) were assessed using the MTT assay. Wound closure was evaluated by wound-healing assay, while cytoskeletal organization and nuclear morphology were examined by phalloidin/DAPI fluorescence microscopy. Apoptosis/necrosis and intracellular reactive oxygen species production were analyzed by flow cytometry using Annexin V-FITC/7-AAD and CM-H_2_DCFDA, respectively. IL-6 release and IL-8 release were quantified by ELISA. **Results:** Both formulations induced concentration-dependent biological effects in hDPSCs. MG-89 showed a higher IC_50_ than MG-85 and preserved metabolic activity, wound closure, cytoskeletal organization, and nuclear morphology more effectively at low concentrations. At higher concentrations, both formulations impaired wound closure and altered cellular morphology, although these effects were more pronounced for MG-85. Flow cytometry showed increased apoptotic/necrotic populations at higher concentrations, particularly after MG-85 exposure. MG-85 also induced greater intracellular ROS production and higher IL-6/IL-8 release than MG-89. **Conclusions:** Within the limitations of this direct-exposure in vitro model, MG-89 exhibited a more favorable cytocompatibility profile than MG-85 in hDPSCs, particularly at low concentrations. These findings support the use of microscopy-based and multiparametric cellular assays for preclinical screening of AgNP-containing dental materials.

## 1. Introduction

Silver has long been used in medicine because of its antimicrobial properties, including applications in wound care, surgical materials, and water purification systems [1]. In dentistry, silver-based compounds such as silver nitrate, silver diamine fluoride, and silver citrate have been investigated for caries management and other preventive or therapeutic applications, although concerns related to staining, concentration-dependent toxicity, and tissue compatibility have limited their broader use [2,3].

The development of nanotechnology has enabled the use of silver in nanoparticulate form. Silver nanoparticles (AgNPs), typically ranging from 1 to 100 nm, exhibit a high surface-area-to-volume ratio and size-dependent biological activity [4]. Their antimicrobial effects have been attributed to several mechanisms, including disruption of bacterial envelopes, induction of oxidative stress, and interaction with intracellular components such as proteins and nucleic acids [5,6]. For this reason, AgNPs have been incorporated into or investigated in a range of dental materials, including adhesives, cements, endodontic sealers, and prosthetic coatings, with the aim of reducing microbial adhesion and biofilm formation [7,8].

Despite these potential advantages, the biological response to AgNP-containing materials remains a critical aspect of preclinical evaluation, particularly when they are intended for dental applications involving dentin or tissues in close proximity to the pulp. Soluble components and nanoscale particles may interact with the dentin–pulp complex, especially under conditions of reduced remaining dentin thickness, and may affect cell viability, wound closure, oxidative balance, and inflammatory signaling [9,10,11,12,13]. Therefore, in vitro cytocompatibility testing using pulp-relevant cell models is essential before further translational development.

Human dental pulp stem cells (hDPSCs) are a relevant cellular model for assessing the biological effects of dental materials because they participate in pulp homeostasis, repair, and dentin–pulp complex regeneration [14]. Previous studies have shown that AgNPs can induce concentration-dependent cytotoxicity in mammalian cells through mechanisms involving oxidative stress, mitochondrial dysfunction, apoptosis, and inflammatory mediator release [15,16,17]. These responses are influenced by several physicochemical parameters, including particle size, morphology, surface charge, coating, aggregation state, and concentration [18,19,20].

This study aimed to comparatively evaluate the in vitro cytocompatibility of two experimental PEI/PVP-stabilized silver nanoparticle formulations, MG-85 and MG-89, in human dental pulp stem cells (hDPSCs). These formulations differ in their reported physicochemical features, including particle size distribution and morphology, which may influence their interaction with biological systems. Using a multiparametric experimental approach that included metabolic activity, wound-healing analysis, apoptosis/necrosis assessment, intracellular reactive oxygen species production, cytokine release, and fluorescence microscopy of cytoskeletal and nuclear morphology, we assessed concentration-dependent cellular responses after exposure to both formulations. The purpose was to identify formulation- and dose-related differences in hDPSC behavior under direct in vitro exposure conditions, providing preclinical information for the biological screening of AgNP-containing dental materials.

## 2. Materials and Methods

### 2.1. Silver Nanoparticle Formulations

The AgNP formulations MG-85 and MG-89 were provided by NanoGap (Santiago de Compostela, Spain). According to the physicochemical characterization data provided by the manufacturer, MG-85 had a mean particle size of 18.83 ± 8.86 nm, as determined by transmission electron microscopy (TEM), and exhibited a quasi-spherical and relatively heterogeneous morphology, whereas MG-89 had a smaller mean particle size of 3.67 ± 1.06 nm and a more regular spherical morphology. The total solid content was 112.7 g/L (Ag@PEI) for MG-85 and 99.3 g/L (Ag@PEI) for MG-89; the total silver content was 26.20 and 16.0 g/L, respectively, and the total PEI content was 86.50 and 83.3 g/L, respectively. Both formulations were PEI-coated, PVP-stabilized, and dispersed in Milli-Q water. These characteristics are summarized in Supplementary Table S1. No additional in-house physicochemical characterization, including DLS, ζ-potential measurements, UV–visible spectroscopy, or silver-ion release analysis, was performed in the present study.

The concentrations used in this study (1, 5, 10, 25, and 50 µg/mL) were calculated based on the nominal amount of each nanoparticle formulation (MG-85 or MG-89), without normalization to their elemental silver content. Therefore, equivalent nominal concentrations of the two formulations did not necessarily correspond to equivalent amounts of silver.

### 2.2. Isolation, Culture and Characterization of Human Dental Pulp Stem Cells (hDPSCs)

The study protocol was approved by the Ethics Committee for Clinical Research of the Universidad de Murcia (ID: M10/2024/176). Human dental pulp stem cells (hDPSCs) were isolated from permanent teeth (n = 10) without caries or pulpal pathology that had been extracted for orthodontic reasons, including premolars and third molars. Deciduous teeth, carious teeth, teeth with pulpal pathology or necrosis, and teeth associated with cystic lesions were excluded. Donor age and sex were not recorded in the anonymized dataset and therefore could not be reported retrospectively. Cells obtained from the ten teeth were pooled after isolation to generate a single hDPSC population, which was subsequently expanded and used throughout the study. All assays were performed using this same pooled cell population at passages 3–5 (P3–P5). The three independent experiments reported for each assay therefore represent separate experimental runs performed with the pooled hDPSC population rather than experiments conducted with cells from different individual donors.

Written informed consent was obtained from all donors prior to sample collection. The samples were anonymized to ensure donor confidentiality. Immediately after extraction, the teeth were placed in 50 mL Falcon tubes containing high-glucose DMEM (Biowest, Nuaillé, France), 10% fetal bovine serum (FBS; Cytiva, Marlborough, MA, USA), 1% antibiotic–antimycotic solution (penicillin G 10,000 U/mL, streptomycin 10,000 U/mL, amphotericin B 25 µg/mL; HyClone, Logan, UT, USA), and stored at 4–8 °C for up to 48 h.

Pulp tissues were retrieved under sterile conditions in a class II biosafety cabinet (Telstar, Terrassa, Spain), using endodontic files (#10 and #15) and barbed broaches. The pulp tissue was enzymatically digested in a solution containing HBSS (Gibco, Grand Island, NY, USA), collagenase type I (200 U/mL; Roche Diagnostics, Mannheim, Germany), and dispase (100 mg/mL stock solution; 5 mg/mL final working concentration; Thermo Fisher Scientific, Waltham, MA, USA) at 37 °C with periodic agitation. Following digestion, cells were centrifuged at 300× *g* for 5 min (Heraeus, Thermo Fisher Scientific), washed, and resuspended in complete growth medium composed of DMEM supplemented with 10% FBS, 1% glutamine, and antibiotics. The cell suspension was seeded in 25 cm^2^ flasks and incubated at 37 °C with 5% CO_2_. After 5–6 days, cell colonies became visible. The culture medium was refreshed every 2 days until 80% confluence was reached.

Cells were harvested by trypsinization using 0.25% Trypsin-EDTA (Sigma-Aldrich, St. Louis, MO, USA). After detachment, cells were centrifuged, resuspended in fresh medium, and counted using the trypan blue exclusion method at a 1:1 ratio (Sigma-Aldrich). Cell viability and concentration were determined using an automated cell counter (TC20™, Bio-Rad, Hercules, CA, USA). The observed cell size ranged from 4 to 34 µm, and cells displayed a spindle-shaped fibroblastoid morphology.

hDPSCs were characterized by flow cytometry according to previously established methods from our group [21]. Briefly, cells were labelled with monoclonal antibodies against CD14, CD20, CD34, CD45, CD73, CD90, and CD105. The cells showed positive expression of the mesenchymal stromal cell markers CD73, CD90, and CD105 and negative expression of the hematopoietic markers CD14, CD20, CD34, and CD45, consistent with a dental pulp-derived mesenchymal stromal cell phenotype.

Cell seeding density was adapted to the plate format and technical requirements of each assay, as specified below, and therefore was not identical across experiments. These densities were not independently optimized as part of the present study.

### 2.3. Determination of the Half-Maximal Inhibitory Concentration (IC_50_)

The half-maximal inhibitory concentration (IC_50_) of the silver nanoparticle formulations MG-89 and MG-85 was determined to evaluate concentration-dependent cytotoxicity and to define working concentrations for subsequent assays. hDPSCs were seeded in 96-well plates at a density of 1500–3000 cells/well in 180 µL of complete growth medium and incubated at 37 °C with 5% CO_2_ for 24 h. Cells were then exposed to serial dilutions of each AgNP formulation ranging from 0.75 to 100 µg/mL for 24 h. Untreated cells cultured in complete growth medium served as controls.

After exposure, MTT reagent was added to the wells at a final concentration of 1 mg/mL and incubated for 4 h at 37 °C. Formazan crystals were solubilized with 100 µL of DMSO (Sigma-Aldrich, USA), and absorbance was measured at 570 nm using an INFINITE 200 PRO microplate reader (Tecan, Grödig, Austria).

IC_50_ values, defined as the concentration of each AgNP formulation required to inhibit 50% of hDPSC metabolic activity, were determined graphically by plotting the percentage of metabolic activity on the *Y*-axis and the AgNP concentration on the *X*-axis. Data were analyzed by non-linear regression using GraphPad Prism software, version 8.10 (GraphPad Software Inc., San Diego, CA, USA and, using the “[inhibitor] vs. response—Variable slope (four parameters)” model, with the experimental concentrations entered directly without logarithmic transformation. The dose–response curves were fitted according to the equation Y = Bottom + (Top − Bottom)/[1 + (X/IC_50_)^HillSlope], where X represents the nanoparticle concentration, Bottom and Top represent the lower and upper plateaus of the fitted curve, respectively, and HillSlope represents the slope factor.

Lower IC_50_ values were interpreted as indicating higher cytotoxicity, whereas higher IC_50_ values indicated greater cellular tolerance under the tested conditions.

Based on the initial dose–response/IC50 assessment, concentrations of 1, 5, 10, 25, and 50 µg/mL were selected for the subsequent experiments to encompass a range of exposures relative to the IC50 values of both formulations. These concentrations were intended for comparative dose–response characterization rather than to reproduce clinically established pulpal exposure levels.

### 2.4. Metabolic Activity Assay

The metabolic activity of hDPSCs after exposure to MG-89 and MG-85 was evaluated using the 3-(4,5-dimethylthiazol-2-yl)-2,5-diphenyltetrazolium bromide (MTT) assay (Sigma-Aldrich, USA). hDPSCs were seeded at a density of 3000 cells/well in 96-well plates and cultured in complete growth medium. After cell adhesion, treatments with MG-89 and MG-85 were applied at concentrations of 1, 5, 10, 25, and 50 µg/mL.

Cells were incubated with the corresponding AgNP concentrations for 24, 48, or 72 h at 37 °C with 5% CO_2_. After each exposure period, MTT reagent was added to the wells at a final concentration of 1 mg/mL and incubated for 4 h at 37 °C. Formazan crystals were solubilized with 100 µL of DMSO (Sigma-Aldrich, USA), and absorbance was measured at 570 nm using an INFINITE 200 PRO microplate reader (Tecan, Austria).

Metabolic activity was expressed as a percentage relative to untreated control cells cultured in complete growth medium. Three independent experimental runs were performed using the same pooled hDPSC population, each carried out in triplicate for each experimental condition.

### 2.5. Wound Closure Assay

A wound healing assay was performed to assess the effect of AgNPs on wound closure. hDPSCs were seeded into 24-well plates and grown to confluence. A linear scratch was made using a sterile pipette tip, and cells were washed and incubated in media containing MG-85 or MG-89 at the indicated dilutions. Images were captured at 0, 24, 48 and 72 h using a phase-contrast microscope. The wound area was quantified using ImageJ software (v1.53e; NIH, Bethesda, MD, USA). Untreated cells cultured in complete medium served as negative controls. All conditions were tested in triplicate to ensure experimental reliability.

### 2.6. Immunofluorescence Assay

Immunofluorescence staining using phalloidin was performed to evaluate possible changes in hDPSC morphology and in the structure and organization of the actin cytoskeleton after exposure to the silver nanoparticle formulations MG-89 and MG-85. hDPSCs were seeded on glass coverslips, allowed to adhere, and cultured in complete growth medium alone as a control or in complete growth medium containing MG-89 or MG-85 at concentrations of 1, 5, 10, 25, and 50 µg/mL for 72 h at 37 °C.

After treatment, cells were washed with PBS, fixed with 4% paraformaldehyde (Merck Millipore, Darmstadt, Germany) in PBS for 20 min, permeabilized with 0.25% Triton X-100 (Sigma-Aldrich, USA) in PBS for 3 min, and stained with Invitrogen™ Alexa Fluor™ 594-conjugated phalloidin (Thermo Fisher Scientific, USA) to visualize F-actin filaments. Cell nuclei were counterstained with 4′,6-diamidino-2-phenylindole dihydrochloride (DAPI; Thermo Fisher Scientific, USA).

Fluorescence micrographs were acquired using a Leica TCS SP2 confocal microscope (Leica, Wetzlar, Germany). Phalloidin staining was used to assess actin filament organization, cell spreading, and cytoskeletal integrity, while DAPI staining was used to evaluate nuclear distribution and morphology. Three independent experiments were performed, each carried out in triplicate for each experimental condition.

### 2.7. Apoptosis/Necrosis Assay

To distinguish between apoptotic and necrotic cell death induced by the silver nanoparticle formulations MG-89 and MG-85, an Annexin V-FITC/7-AAD flow cytometry assay was performed. hDPSCs were seeded in 6-well plates at a density of 100,000 cells/well in 2 mL of DMEM, treated with the corresponding AgNP concentrations, and incubated for 72 h at 37 °C. After treatment, both adherent cells and culture supernatants were collected to include detached cells. Cells were then stained with Annexin V-FITC and 7-AAD (Immunostep, Salamanca, Spain) according to the manufacturer’s instructions.

Samples were analyzed by flow cytometry using a BD FACSCanto™ cytometer, and data were processed with FlowJo software(v10.9). Results were presented as quadrant dot plots: viable cells were identified as Annexin V-negative/7-AAD-negative, early apoptotic cells as Annexin V-positive/7-AAD-negative, late apoptotic cells as Annexin V-positive/7-AAD-positive, and necrotic cells as Annexin V-negative/7-AAD-positive.

### 2.8. Intracellular Reactive Oxygen Species (ROS) Assay

Intracellular reactive oxygen species (ROS) production was evaluated in hDPSCs using the fluorescent probe CM-H_2_DCFDA. Cells were cultured in 6-well plates until approximately 80% confluence and then exposed to MG-89 or MG-85 at concentrations of 1, 5, 10, 25, and 50 µg/mL for 24 h. Untreated cells cultured in complete medium were used as controls.

After treatment, cells were detached using TrypLE Express (Thermo Fisher Scientific), washed twice with PBS, and incubated with 5 µM CM-H_2_DCFDA (Molecular Probes, Eugene, OR, USA) in PBS for 30 min at 37 °C in the dark. Fluorescence was measured by flow cytometry using an LSR Fortessa X-20 cytometer (BD Biosciences, San Jose, CA, USA), with excitation/emission wavelengths of 492/517 nm. Data were analyzed using FlowJo software to determine the percentage of ROS-positive cells under each experimental condition. All assays were performed in triplicate.

### 2.9. ELISA

A sandwich enzyme-linked immunosorbent assay (ELISA) was used to quantify the proinflammatory cytokines IL-6 and IL-8 in hDPSC culture supernatants following exposure to MG-89 and MG-85. Cells were seeded in 24-well plates, treated with the corresponding AgNP concentrations, and incubated for 72 h at 37 °C. After treatment, culture supernatants were collected, centrifuged to remove cellular debris, and stored or processed according to the manufacturer’s instructions.

IL-6 and IL-8 concentrations were quantified using commercial FineTest ELISA kits (FineTest Biotech Inc., Boulder, CO, USA), following the manufacturer’s protocol. Briefly, samples and standards were added to wells pre-coated with capture antibodies, followed by incubation with biotinylated detection antibodies, streptavidin–HRP conjugate, and TMB substrate. The enzymatic reaction was stopped with 1 M sulfuric acid, and absorbance was measured at 450 nm using a microplate reader (INFINITE 200 PRO, Tecan, Austria). Cytokine concentrations were calculated from standard curves and analyzed using GraphPad Prism software. IL-6 and IL-8 concentrations were expressed as pg/mL of culture supernatant and were not normalized to viable cell number or total cellular protein. All assays were performed in triplicate.

### 2.10. Statistical Analysis

Statistical analysis was performed using GraphPad Prism v8.10 (GraphPad Software). Data were checked for normality using Q–Q plots and expressed as mean ± standard deviation (SD). For each assay and at each tested concentration, differences among untreated control cells, MG-85-treated cells, and MG-89-treated cells were analyzed using one-way ANOVA followed by Tukey’s post hoc test for multiple comparisons. Each concentration was analyzed independently. A *p*-value < 0.05 was considered statistically significant. Significance levels were indicated in figures as *p* < 0.05 (*), *p* < 0.01 (**), and *p* < 0.001 (***).

## 3. Results

### 3.1. IC_50_ Analysis

**Figure 1:**
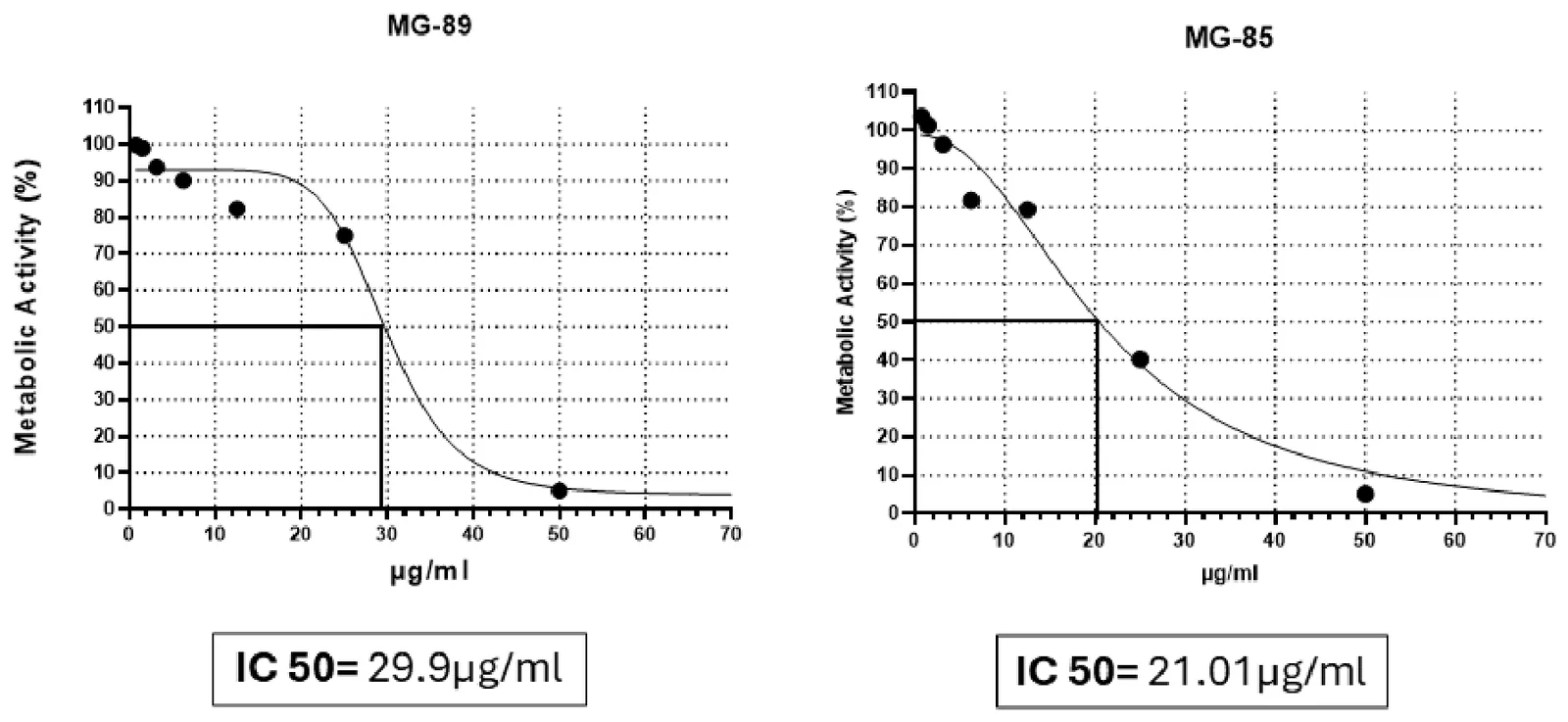
Dose–response curves for MG-89 and MG-85 showing IC50 values. The sigmoidal curves represent metabolic activity (%) as a function of nanoparticle concentration (µg/mL). The horizontal line indicates 50% metabolic activity, and the vertical line indicates the corresponding IC50 value on the concentration axis.

The dose–response curves showed different cytotoxicity profiles for the two AgNP formulations. The estimated IC_50_ value was 29.9 µg/mL for MG-89 and 21.01 µg/mL for MG-85. These results indicate that, under the tested direct-exposure conditions, hDPSCs tolerated higher concentrations of MG-89 than MG-85 before reaching a 50% reduction in metabolic activity (Figure 1).

### 3.2. Metabolic Activity Analysis

**Figure 2:**
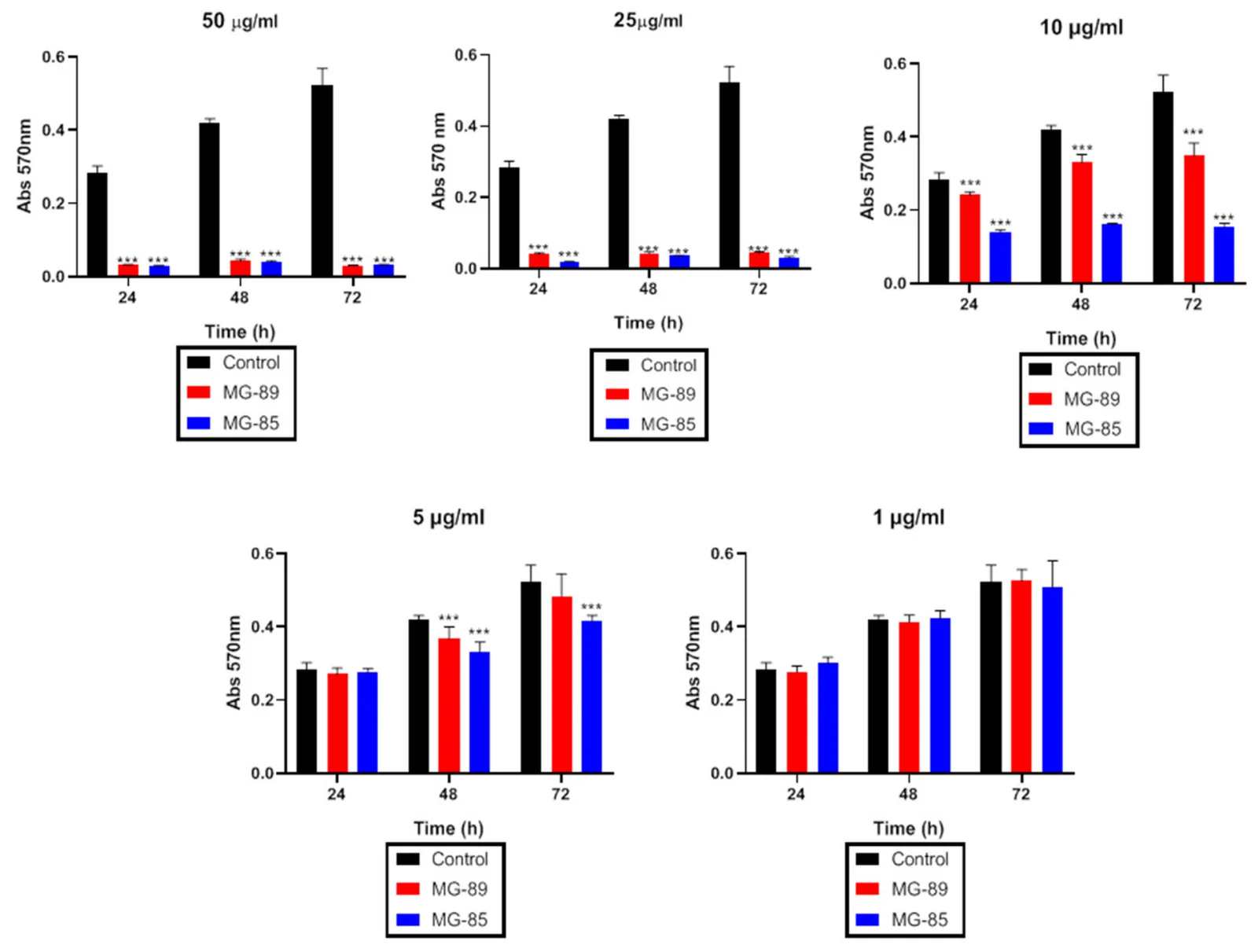
Metabolic activity of hDPSCs treated with MG-89 and MG-85 at different concentrations (1, 5, 10, 25, and 50 µg/mL) over 24, 48, and 72 h. Cell viability was measured by MTT assay and is expressed as percentage relative to untreated control. Bars represent mean ± SD from three independent experiments. Asterisks indicate statistical significance compared to control (*** *p* < 0.001). Significant reductions in metabolic activity were observed at concentrations ≥10 µg/mL, particularly with MG-85.

The metabolic activity of hDPSCs treated with MG-89 and MG-85 was assessed at 24, 48, and 72 h using the MTT assay at five concentrations: 1, 5, 10, 25, and 50 µg/mL. Both formulations produced a concentration- and time-dependent reduction in metabolic activity (Figure 2).

At the highest concentrations, 25 and 50 µg/mL, both MG-89 and MG-85 significantly reduced metabolic activity from the earliest time point (*** *p* < 0.001 vs. control). At 10 µg/mL, metabolic activity was partially preserved, although values remained significantly lower than those of untreated control cells (*** *p* < 0.001). At 5 µg/mL, MG-89 showed a less pronounced reduction in metabolic activity than MG-85, whereas at 1 µg/mL neither formulation produced significant changes compared with the untreated control.

Overall, MG-89 showed a more favorable metabolic activity profile than MG-85 at lower concentrations, while both formulations showed marked cytotoxic effects at higher concentrations.

### 3.3. Wound Closure Assay

**Figure 3:**
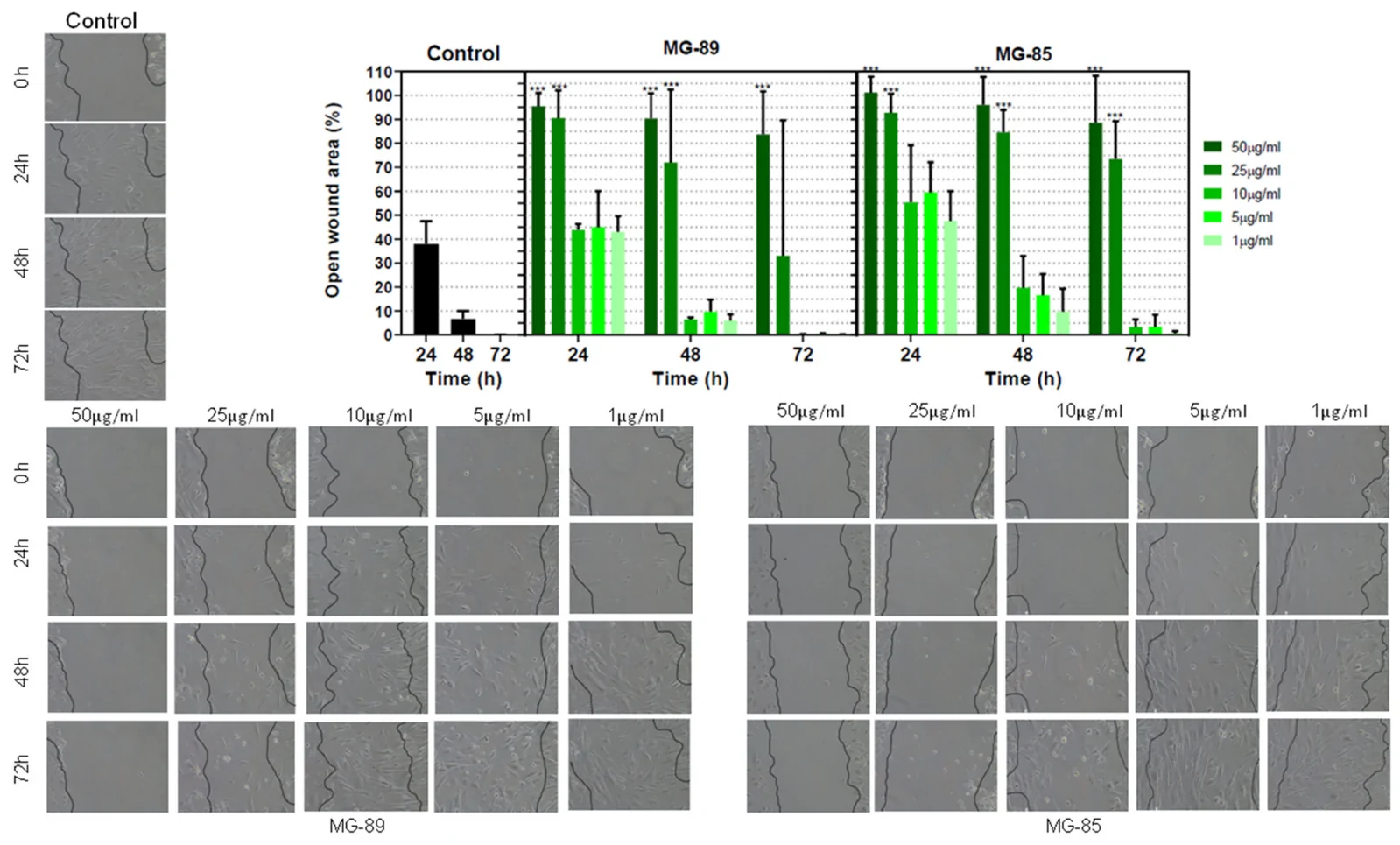
Quantitative and qualitative analysis of hDPSC wound closure following treatment with MG-89 and MG-85 at varying concentrations. Percentage of wound closure is shown at 24, 48, and 72 h. Representative images and bar graphs indicate impaired wound closure at higher concentrations (50 and 25 µg/mL), particularly for MG-85, and improved closure at lower concentrations. Asterisks indicate statistically significant differences versus control (*** *p* < 0.001).

A wound-healing assay was performed to evaluate the effect of MG-89 and MG-85 on hDPSC wound closure over 72 h. Differences were observed between formulations and across concentrations (Figure 3).

MG-85 markedly impaired wound closure at 50 and 25 µg/mL, with limited closure even after 72 h. Cells exposed to these concentrations also showed reduced confluence and altered morphology. At 10 µg/mL, partial wound closure was observed by 72 h, whereas 5 and 1 µg/mL showed wound-closure patterns closer to those of untreated control cells.

MG-89 also reduced wound closure at the highest concentrations, particularly at 50 and 25 µg/mL, although some initial closure was observed during the first 24 h. At 10 µg/mL, the wound-closure pattern was closer to the control condition than at higher concentrations. The most favorable response was observed at 1 µg/mL, with more homogeneous cell distribution and greater wound closure.

Quantitative analysis showed a concentration-dependent reduction in wound closure, with significant impairment at the highest concentrations compared with the control (*** *p* < 0.001). Overall, MG-89 preserved wound-closure capacity more effectively than MG-85 at lower concentrations, whereas both formulations impaired wound closure at higher concentrations.

### 3.4. Immunofluorescence Analysis

**Figure 4:**
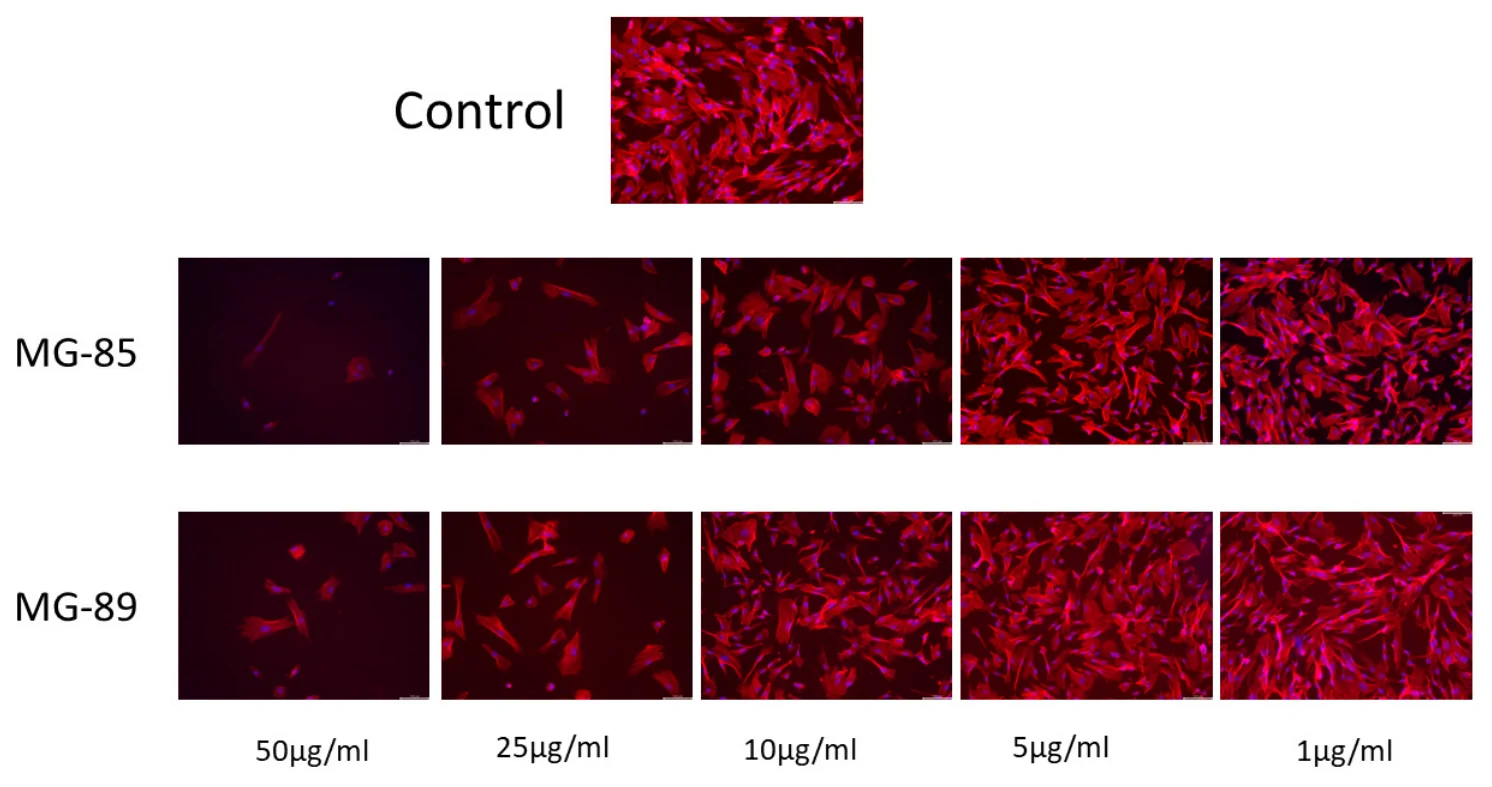
Immunofluorescence images of hDPSCs treated with MG-85 and MG-89 at various concentrations, compared to the untreated control. Actin filaments were stained with phalloidin (red) and nuclei with DAPI (blue). High concentrations (50 and 25 µg/mL) disrupted cytoskeletal and nuclear structure, while lower concentrations (5 and 1 µg/mL) preserved normal morphology, particularly in MG-89-treated cells.

Fluorescence microscopy revealed concentration-dependent changes in cytoskeletal organization and nuclear morphology after exposure to MG-85 and MG-89 (Figure 4). Untreated control cells showed a well-organized actin cytoskeleton, preserved nuclear morphology, and homogeneous cell distribution.

At 50 and 25 µg/mL, both formulations were associated with reduced cell density, altered cell spreading, disruption of F-actin organization, and abnormal nuclear morphology. These changes were more evident in MG-85-treated cells. At 10 µg/mL, MG-89-treated cells showed partial preservation of cytoskeletal organization and nuclear distribution, whereas MG-85-treated cells still showed more evident morphological alterations.

At the lowest concentrations, 5 and 1 µg/mL, cellular morphology was better preserved, particularly in the MG-89 group, with actin organization and nuclear morphology closer to those observed in untreated control cells. Overall, the fluorescence microscopy findings were consistent with the metabolic activity and wound-healing results, showing a more favorable cellular morphology profile for MG-89 at lower concentrations.

### 3.5. Apoptosis/Necrosis Analysis

**Figure 5:**
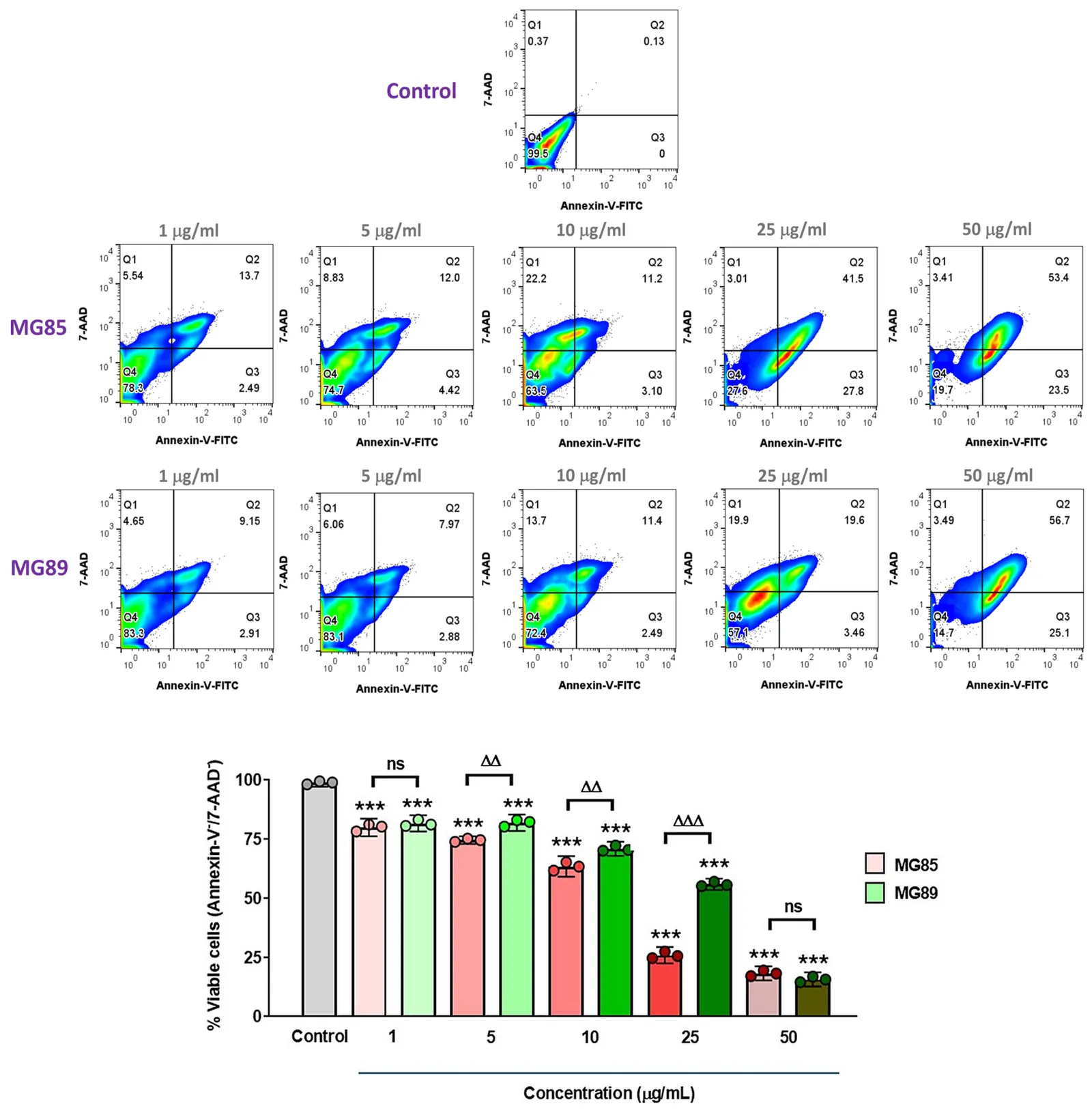
Representative Annexin V-FITC/7-AAD flow cytometry plots showing viability, apoptosis, and necrosis profiles of hDPSCs exposed to MG-85 and MG-89 at concentrations ranging from 1 to 50 µg/mL. Quadrants correspond to Q4 (viable cells, Annexin V^−^/7-AAD^−^), Q3 (early apoptotic cells, Annexin V^+^/7-AAD^−^), Q2 (late apoptotic cells, Annexin V^+^/7-AAD^+^), and Q1 (necrotic cells, Annexin V^−^/7-AAD^+^). Representative dot plots from one experiment are shown. The lower panel shows the percentage of viable cells (Q4) from three independent experiments (mean ± SD, n = 3). Cell viability decreased in a concentration-dependent manner for both formulations, with significantly higher viability observed in MG-89-treated cells than in MG-85-treated cells at 5, 10, and 25 µg/mL. Asterisks indicate significant differences compared with the untreated control group (*** *p* < 0.001), whereas Δ symbols indicate significant differences between MG-85 and MG-89 at the same concentration (^ΔΔ^ *p* < 0.01, ^ΔΔΔ^ *p* < 0.001; ns, not significant).

Flow cytometry analysis using Annexin V-FITC/7-AAD staining was performed to evaluate the distribution of viable, apoptotic, and necrotic hDPSCs after exposure to MG-85 and MG-89 (Figure 5). Untreated control cells showed a predominant viable cell population.

For MG-85, exposure to 50 µg/mL resulted in a marked reduction in viable cells, with a predominant population of late apoptotic cells (53.4%) and lower proportions of early apoptotic cells (23.5%) and viable cells (19.7%). At 25 µg/mL, late apoptotic cells remained the most represented population, although their percentage decreased to 41.5%. At 10 µg/mL, the distribution shifted toward a higher proportion of viable cells, although apoptotic and necrotic populations were still observed. At 5 and 1 µg/mL, more than 75% of MG-85-treated cells remained viable.

MG-89 showed a concentration-dependent response. At 50 µg/mL, the viable cell population was reduced to 14.7%. At 25 µg/mL, viability increased to 57.1%, although necrotic cells represented nearly 20% of the population. At 10 µg/mL, viability increased to 72.4%, and at 5 and 1 µg/mL more than 83% of cells remained viable, with lower proportions of apoptotic or necrotic cells.

Overall, both formulations induced concentration-dependent changes in cell death profiles. At lower and intermediate concentrations (1–25 μg/mL), MG-89 was generally associated with a higher proportion of viable cells than MG-85.

We thank the reviewer for this comment. To address this concern, Figure 5 has been revised to include a quantitative summary of the apoptosis assay results from all three independent experiments (n = 3). The new graph shows the percentage of viable cells (Annexin V^−^/7-AAD^−^, Q4) as mean ± SD, together with the individual experimental values. In addition, direct statistical comparisons between MG-85 and MG-89 were performed at each concentration. MG-89 showed significantly higher viability than MG-85 at 5, 10, and 25 µg/mL, whereas no significant differences were observed at 1 or 50 µg/mL. The figure legend, Results, and Discussion sections have been updated accordingly.

### 3.6. Intracellular ROS Production

**Figure 6:**
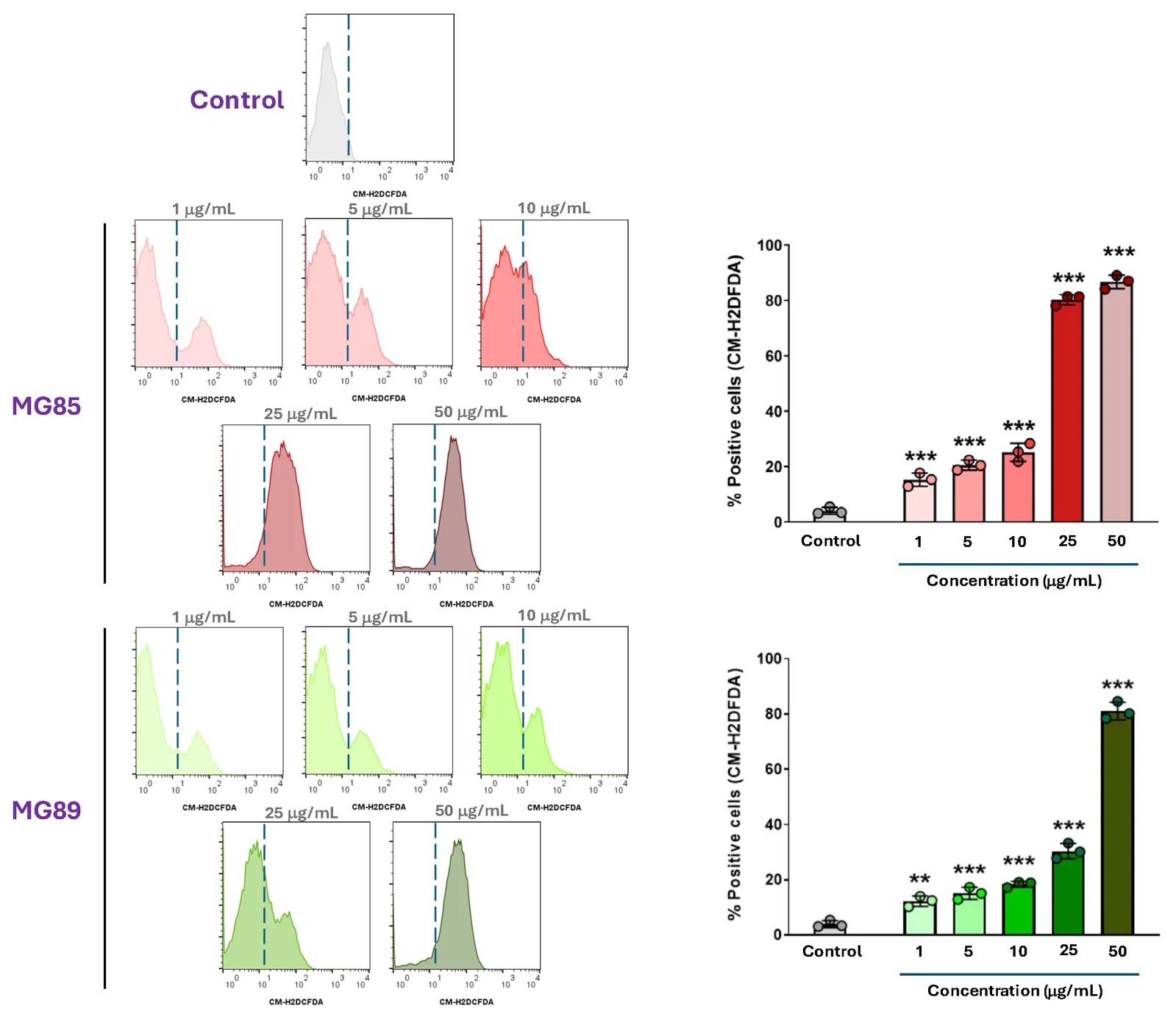
Intracellular ROS production in hDPSCs treated with MG-85 and MG-89, assessed using CM-H_2_DCFDA fluorescence. Representative flow-cytometry histograms are shown together with the quantitative analysis of CM-H_2_DCFDA-positive cells at the different concentrations tested. Both treatments significantly increased ROS production compared with the untreated control, with MG-85 inducing higher ROS levels across the tested concentrations. Bars represent mean ± SD from three independent experiments. Asterisks indicate statistical significance compared with control (** *p* < 0.01, *** *p* < 0.001).

Intracellular reactive oxygen species (ROS) production was evaluated by flow cytometry using the CM-H_2_DCFDA probe (Figure 6). Both MG-85 and MG-89 increased the percentage of ROS-positive cells compared with untreated control cells, although the magnitude of this response differed between formulations and concentrations.

MG-85 induced a marked increase in ROS-positive cells across the concentration range tested, with the highest values observed at concentrations of 25 and 50 µg/mL. Increased ROS production was also detected at lower concentrations. In contrast, MG-89 produced a more gradual increase in ROS-positive cells, remaining below 40% up to 25 µg/mL. At 50 µg/mL, ROS production increased sharply and reached values closer to those observed for MG-85.

Overall, MG-85 was associated with a stronger oxidative response than MG-89 under the tested conditions, whereas MG-89 showed a lower ROS-positive cell percentage at low and intermediate concentrations.

### 3.7. ELISA for IL-6 and IL-8 Quantification

**Figure 7:**
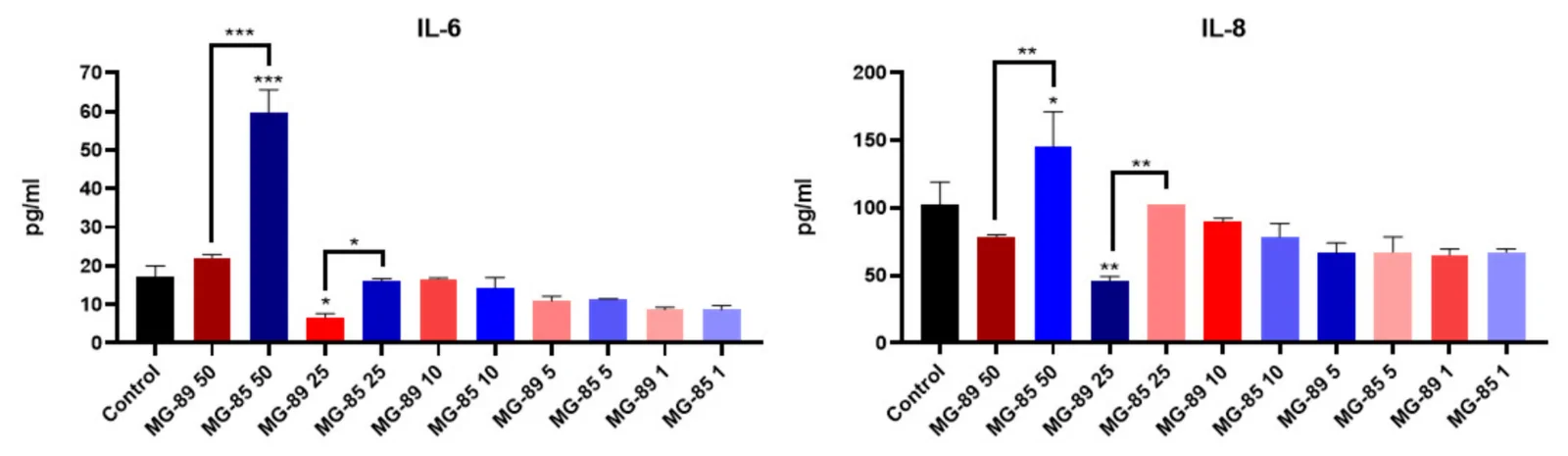
Quantification of IL-6 and IL-8 secretion by hDPSCs after treatment with MG-85 and MG-89 at various concentrations, assessed by ELISA. Bar graphs represent cytokine levels (pg/mL) for IL-6 and IL-8. MG-85 at 50 µg/mL significantly increased both IL-6 and IL-8 levels compared to control and to MG-89 (*** *p* < 0.001 for IL-6; ** *p* < 0.01 for IL-8). MG-89 at 25 µg/mL showed significantly lower cytokine secretion compared to control and MG-85 (* *p* < 0.05 or ** *p* < 0.01), indicating a lower IL-6/IL-8 secretion profile under the tested experimental conditions. Bars represent mean ± SD from three independent experiments. Asterisks indicate statistical significance versus control.

ELISAs were performed to evaluate IL-6 and IL-8 secretion by hDPSCs after exposure to MG-85 and MG-89 at different concentrations (Figure 7). For IL-6, MG-85 at 50 µg/mL induced a marked increase compared with both untreated control cells and MG-89 at the same concentration (*** *p* < 0.001). At 25 µg/mL, MG-89 showed significantly lower IL-6 levels than both untreated control cells and MG-85 at the same concentration (* *p* < 0.05). At the remaining concentrations, IL-6 levels were comparable to or lower than control values, without statistically significant differences.

For IL-8, MG-85 at 50 µg/mL significantly increased cytokine secretion compared with untreated control cells (* *p* < 0.05), and IL-8 levels were significantly higher than those observed for MG-89 at the same concentration (** *p* < 0.01). At 25 µg/mL, MG-89 showed significantly lower IL-8 secretion than both untreated control cells and MG-85 (** *p* < 0.01).

Overall, MG-85 induced a stronger cytokine release profile at the highest concentration tested, whereas MG-89 was associated with lower IL-6 and IL-8 secretion under the same experimental conditions.

## 4. Discussion

This study comparatively evaluated the in vitro biological response of human dental pulp stem cells (hDPSCs) exposed to two experimental PEI/PVP-stabilized silver nanoparticle formulations, MG-85 and MG-89. A multiparametric approach was used, including metabolic activity, wound-healing analysis, fluorescence microscopy of cytoskeletal and nuclear morphology, apoptosis/necrosis assessment, intracellular ROS production, and IL-6/IL-8 quantification. Overall, both formulations induced concentration-dependent effects, with MG-89 showing a more favorable cytocompatibility profile than MG-85 under the tested direct-exposure conditions, particularly at lower concentrations. These findings should be interpreted as preclinical in vitro evidence of formulation-dependent cellular responses, rather than as direct evidence of clinical safety or antimicrobial efficacy.

The IC_50_ and MTT results showed a concentration-dependent reduction in hDPSC metabolic activity after exposure to both AgNP formulations. MG-89 showed a higher IC_50_ value than MG-85, indicating that hDPSCs tolerated higher concentrations of MG-89 before reaching a 50% reduction in metabolic activity. This pattern was also reflected in the time-dependent MTT assay, where MG-89 preserved metabolic activity more effectively than MG-85 at lower concentrations, particularly at 1 and 5 µg/mL. These findings are consistent with previous studies reporting dose-dependent cytotoxic effects of AgNPs in mesenchymal stem cells and other mammalian cell models [22,23,24,25]. However, since MTT primarily reflects mitochondrial/metabolic activity, these results should be interpreted together with complementary assays assessing morphology, cell death, oxidative stress, and inflammatory mediator release.

The wound-healing assay showed that both AgNP formulations affected hDPSC wound closure in a concentration-dependent manner, with a more pronounced impairment observed after MG-85 exposure. At lower concentrations, particularly 1 and 5 µg/mL, MG-89-treated cells showed wound-closure patterns closer to those of untreated control cells. Since no proliferation inhibitor was used in this assay, wound closure should be interpreted as the combined result of wound closure and proliferation rather than as a pure measure of migratory capacity. Nevertheless, this assay provides relevant information on the functional behavior of hDPSCs after exposure to the tested formulations, as wound closure is closely related to cell spreading, cytoskeletal integrity, and proliferative capacity. These findings are in agreement with Vieira et al., who reported that metallic nanoparticles may reduce human fibroblast wound closure in vitro [26].

Fluorescence microscopy provided complementary morphological evidence supporting the concentration-dependent effects observed in the metabolic activity and wound-healing assays. At higher concentrations, particularly 25 and 50 µg/mL, both formulations were associated with reduced cell density, altered cell spreading, disruption of F-actin organization, and abnormal nuclear morphology. These alterations were more evident after MG-85 exposure, whereas MG-89-treated cells showed better preservation of cytoskeletal organization and nuclear morphology at lower concentrations. Since F-actin organization is closely related to cell adhesion, spreading, and wound closure, the morphological changes observed by fluorescence microscopy may help explain the impaired wound-closure response observed at higher concentrations. Previous studies have associated AgNP-induced cytotoxicity with oxidative stress, mitochondrial injury, and apoptotic signaling in mammalian cell models [27,28].

The apoptosis/necrosis analysis further supported the concentration-dependent cytotoxic profile of both AgNP formulations. Flow cytometry showed a reduction in viable hDPSCs and an increase in apoptotic and/or necrotic populations at higher concentrations, particularly after exposure to MG-85. In contrast, MG-89 maintained a higher proportion of viable cells at lower concentrations, especially at 1 and 5 µg/mL. Quantitative analysis of three independent experiments showed significantly higher percentages of viable cells in MG-89-treated cultures than in MG-85-treated cultures at 5, 10, and 25 µg/mL, whereas no significant differences were observed at 1 or 50 µg/mL. These findings are consistent with previous reports showing that AgNPs and silver ions can induce apoptosis and necrosis in mammalian cells in a concentration- and surface chemistry-dependent manner [23]. The observed cell death profile may also be related to intracellular stress mechanisms associated with AgNP exposure, including the so-called “Trojan horse” mechanism described by Park et al., in which internalized AgNPs may contribute to intracellular silver ion release and subsequent cytotoxic effects [17]. However, because silver ion release and nanoparticle internalization were not directly assessed in the present study, this mechanism should be considered a possible explanation rather than a demonstrated pathway.

Reactive oxygen species production is one of the mechanisms commonly associated with AgNP-induced cytotoxicity. In the present study, MG-85 induced a stronger increase in ROS-positive hDPSCs than MG-89, particularly at intermediate and high concentrations. MG-89 showed a more gradual oxidative response, with lower percentages of ROS-positive cells at low and intermediate concentrations. These findings are consistent with previous studies reporting that AgNPs can increase intracellular ROS production in mammalian cells, contributing to oxidative stress and reduced cell viability [29,30,31]. Nevertheless, ROS production was assessed at 24 h, whereas other endpoints, such as cytokine release and apoptosis/necrosis, were evaluated at later time points. Therefore, the relationship between ROS generation and subsequent cellular responses should be interpreted cautiously and should not be considered direct mechanistic evidence in the absence of intermediate time-point analyses.

The ELISA results showed that MG-85 induced higher IL-6 and IL-8 release than MG-89 at the highest concentration tested, whereas MG-89 was associated with lower cytokine secretion under the same experimental conditions. IL-6 and IL-8 are relevant inflammatory mediators in the dental pulp and have been reported to be increased in inflamed pulp tissues [32]. Therefore, the lower cytokine release observed after MG-89 exposure may be interpreted as part of its more favorable in vitro cellular response profile compared with MG-85. However, because no positive inflammatory stimulus, such as lipopolysaccharide or TNF-α, was included, these findings should not be interpreted as evidence of a specific anti-inflammatory effect. Instead, they indicate that MG-89 induced a lower IL-6/IL-8 secretion profile than MG-85 in hDPSCs under the tested direct-exposure conditions. In addition, cytokine concentrations were not normalized to viable cell number or total cellular protein. Given the concentration-dependent cytotoxicity observed for both formulations, particularly at higher concentrations, differences in IL-6 and IL-8 levels may partly reflect differences in the number of viable cytokine-producing cells. Therefore, these findings should be interpreted as differences in cytokine concentrations in the culture supernatant rather than as differences in cytokine secretion on a per-cell basis.

Differences in the cellular responses observed between MG-85 and MG-89 may be partly related to the physicochemical characteristics reported for each formulation. According to the information provided by the nanoparticle developer, MG-85 displayed a more heterogeneous profile, whereas MG-89 presented a more regular morphology, as summarized in Supplementary Table S1. Previous studies have shown that nanoparticle size, morphology, coating, surface chemistry, and aggregation state can influence cellular uptake, oxidative stress, and cytotoxicity [16,17,30,33]. However, in the present study, the physicochemical characterization was based on data provided by the manufacturer, and additional in-house analyses such as dynamic light scattering in culture medium, ζ-potential measurements, silver ion release, or cellular uptake were not performed. Therefore, any interpretation linking the biological differences between MG-85 and MG-89 to specific physicochemical features should be considered hypothesis-generating rather than mechanistically demonstrated.

The differences observed in metabolic activity, wound closure, cytoskeletal organization, ROS production, apoptosis/necrosis, and cytokine release suggest that MG-89 induced a less disruptive cellular response than MG-85 in hDPSCs, particularly at low concentrations. From a preclinical perspective, preservation of hDPSC viability, morphology, and functional behavior is relevant when evaluating dental materials that may interact with the dentin–pulp complex. However, the present study was based on a direct-exposure in vitro model and did not include dentin barrier systems, pulp chamber models, or antimicrobial assays. Therefore, the results cannot be used to establish clinical safety, dentin diffusion behavior, or disinfectant efficacy. Future studies should evaluate these formulations using dentin disc or pulp chamber models, together with antimicrobial testing against clinically relevant oral microorganisms, before any translational application can be proposed.

A strength of this study is the use of a multiparametric experimental approach to evaluate the response of hDPSCs to two AgNP formulations. The combination of metabolic activity assays, wound-healing analysis, fluorescence microscopy, apoptosis/necrosis assessment, ROS quantification, and cytokine measurement allowed the evaluation of complementary aspects of cellular behavior. In particular, fluorescence microscopy provided morphological information on cytoskeletal organization, cell spreading, and nuclear preservation, supporting the interpretation of the functional and cytotoxicity assays. This integrated approach is useful for preclinical screening of AgNP-containing dental materials, although it does not replace more complex translational models.

Several limitations should be considered when interpreting these findings. First, this study was performed using a direct-exposure in vitro model, which allows controlled comparison between formulations but does not fully reproduce the complexity of the dentin–pulp interface. Second, clinically relevant concentrations of these formulations reaching the dental pulp have not been established. Therefore, the concentration range evaluated in this study should be interpreted as an experimental dose–response range rather than as a simulation of clinical pulpal exposure. Third, the physicochemical information of the AgNP formulations was based on data provided by the nanoparticle developer, and further characterization under cell-culture conditions would help clarify the relationship between nanoparticle properties and biological response. An additional limitation is the absence of a stabilizer-only control containing PEI/PVP without silver nanoparticles. Consequently, the contribution of the stabilizing agents to the observed cellular responses cannot be distinguished from that of the silver nanoparticle formulations themselves, and the present findings should therefore be interpreted as responses to the complete MG-85 and MG-89 formulations rather than as effects attributable exclusively to the silver nanoparticles. An additional limitation is that the tested concentrations were not normalized to the silver content of each formulation, which may have influenced the observed cytotoxicity. Furthermore, silver ion release was not assessed, limiting the interpretation of differences between MG-85 and MG-89. In addition, cytokine concentrations were not normalized to viable cell number or total cellular protein. This limits the interpretation of differences in IL-6 and IL-8 concentrations between formulations, particularly at higher nanoparticle concentrations where substantial cytotoxicity was observed. Finally, the present work focused on short-term cellular responses in hDPSCs. Future studies using dentin barrier models, longer exposure periods, more detailed physicochemical characterization under experimental conditions, and complementary antimicrobial assays are therefore needed to further assess the translational potential of these formulations.

Despite these limitations, the present findings highlight the relevance of formulation-dependent cellular responses when evaluating AgNP-containing materials in pulp-related cell models. MG-89 showed a more favorable in vitro profile than MG-85 across several complementary endpoints, particularly at low concentrations, including metabolic activity, wound closure, cytoskeletal organization, oxidative response, cell death distribution, and cytokine release. These results support the usefulness of multiparametric cellular and microscopy-based approaches for the preliminary biological screening of AgNP formulations intended for further dental materials research.

## 5. Conclusions

MG-85 and MG-89 induced concentration-dependent effects on human dental pulp stem cells under direct in vitro exposure conditions. Under the experimental conditions evaluated, MG-89 showed a more favorable in vitro cellular response than MG-85, particularly at low concentrations, with better preservation of metabolic activity, wound-closure capacity, cytoskeletal organization, nuclear morphology, and cell viability, together with lower ROS-positive cell percentages and lower IL-6/IL-8 concentrations in culture supernatants. These findings describe the cellular response to the two supplied formulations under the tested conditions and do not establish which specific nanoparticle property or formulation characteristic was responsible for the observed differences. These findings are limited to the present in vitro model and should not be interpreted as evidence of greater clinical safety of MG-89. Further studies using dentin barrier models, extended exposure periods, and antimicrobial assays are required before the translational or clinical relevance of these differences can be established.

## Data Availability

The data supporting the findings of this study are available from the corresponding author upon reasonable request.

## Supplementary Materials

The following supporting information can be downloaded at: https://www.mdpi.com/article/10.3390/dj14100682/s1, Table S1: Physicochemical characteristics of MG-85 and MG-89 provided by the manufacturer.

## Abbreviations

7-AAD, 7-aminoactinomycin D; AgNPs, silver nanoparticles; ANOVA, analysis of variance; BD, Becton Dickinson; CD, cluster of differentiation; CM-H_2_DCFDA, chloromethyl-2′,7′-dichlorodihydrofluorescein diacetate; CO_2_, carbon dioxide; DAPI, 4′,6-diamidino-2-phenylindole; DMEM, Dulbecco’s modified Eagle medium; DMSO, dimethyl sulfoxide; EDTA, ethylenediaminetetraacetic acid; ELISA, enzyme-linked immunosorbent assay; EU, European Union; F-actin, filamentous actin; FBS, fetal bovine serum; FITC, fluorescein isothiocyanate; FSRM, Fundación Séneca–Science and Technology Agency of the Region of Murcia; HBSS, Hank’s balanced salt solution; hDPSCs, human dental pulp stem cells; HRP, horseradish peroxidase; IC_50_, half-maximal inhibitory concentration; ID, identifier; IDIS, Health Research Institute of Santiago de Compostela; IL-6, interleukin-6; IL-8, interleukin-8; IMIB, Biomedical Research Institute of Murcia; ISCIII, Instituto de Salud Carlos III; JNK, c-Jun N-terminal kinase; MG-85, experimental silver nanoparticle formulation 85; MG-89, experimental silver nanoparticle formulation 89; MTT, 3-(4,5-dimethylthiazol-2-yl)-2,5-diphenyltetrazolium bromide; NIH, National Institutes of Health; OMEQUI, Medical-Surgical Dentistry Research Group; PBS, phosphate-buffered saline; PEI, polyethyleneimine; PVP, polyvinylpyrrolidone; Q–Q plot, quantile–quantile plot; RD24/0014/0040, RICORS project reference number; RICORS, Redes de Investigación Cooperativa Orientadas a Resultados en Salud; ROS, reactive oxygen species; SD, standard deviation; SP2, spectral confocal microscope model 2; TC20, automated cell counter model TC20; TERAV, Spanish Network of Advanced Therapies; TMB, 3,3′,5,5′-tetramethylbenzidine; TNF-α, tumor necrosis factor alpha; USC, University of Santiago de Compostela; UV–visible spectroscopy, ultraviolet–visible spectroscopy; ζ-potential, zeta potential.
