# Supplementary material for: Comparative Cytocompatibility and Morphological Assessment of Silver Nanoparticle Formulations in Human Dental Pulp Stem Cells
Source: Dent J (Basel). 2026 Oct 19;14(10):682. doi: 10.3390/dj14100682 (PMC13702956; doi:10.3390/dj14100682)
Supplement: Supplementary file 1 [file dentistry-14-00682-s001.zip › dentistry-4556249-supplementary.pdf]

Supplementary Table S1. Physicochemical characteristics of MG-85 and MG-89 provided by the manufacturer.

Parameter	MG-85	MG-89
Total solid content	112.7 g/L (Ag@PEI)	99.3 g/L (Ag@PEI)
Total silver content	26.20 g/L (Ag)	16.0 g/L (Ag)
Total PEI content	86.50 g/L (PEI)	83.3 g/L (PEI)
Mean particle size determined by TEM	18.83 ± 8.86 nm	3.67 ± 1.06 nm
Morphology	Quasi-spherical	Spherical
Coating	PEI	PEI
Stabilizer	PVP	PVP
Dispersion medium	Milli-Q water	Milli-Q water

Note. Data were provided by the manufacturer. No independent physicochemical characterization of the formulations was performed as part of the present study. Silver-ion release data were not available.

Ag, silver; PEI, polyethyleneimine; PVP, polyvinylpyrrolidone; TEM, transmission electron microscopy.
